# Supplementary material for: Content-rich biological network constructed by mining PubMed abstracts
Source: BMC Bioinformatics. 2004 Oct 8;5:147. doi: 10.1186/1471-2105-5-147 (PMC528731; doi:10.1186/1471-2105-5-147)
Supplement: Additional File 5 — The original Chilibot query results of the term "long-term potentiation (LTP)" and 22 other terms, limiting the latest references analyzed to the years 1990, 1995, 2000, and 2004. [file 1471-2105-5-147-S5.bz2 › chilibotAdditionalFile5/ltp2000/html/TRKB_TAU.html]

 


 **TRKB** and **TAU** 
  
Found 3 abstracts in PubMed,  **3 abstracts were retrieved and analyzed**.  


---

 Search Google  |
 PDF files only 
|  EDU domain only 

---

**Interactive relationship** (e.g. stimulation, inhibition, etc)

- A floxed  **trkB**  allele was designed so that neurons lacking  **TrkB**  express  **tau**  beta galactosidase.  Ref: 10798407 Neuron, 2000
- We find that BDNF but not NGF rapidly upregulates levels of synaptophysin,  **tau**  and c Fos correlating with expression of the neurotrophin receptor  **TrkB** .  Ref: 9185679 Neurosci Lett, 1997

**Parallel relationship** (e.g. studied together, co-existance, homology, etc.)

- Brain derived neurotrophic factor induces a rapid upregulation of synaptophysin and  **tau**  proteins via the neurotrophin receptor  **TrkB**  in rat cerebellar granule cells.We have examined the effects of neurotrophins brain derived neurotrophic factor and nerve growth factor on the expression of the maturation specific proteins synaptophysin and  **tau** , and the growth associated protein 43 in cerebellar granule cells.  Ref: 9185679 Neurosci Lett, 1997
